# Development and mapping of Simple Sequence Repeat markers for pearl millet from data mining of Expressed Sequence Tags

**DOI:** 10.1186/1471-2229-8-119

**Published:** 2008-11-27

**Authors:** S Senthilvel, B Jayashree, V Mahalakshmi, P Sathish Kumar, S Nakka, T Nepolean, CT Hash

**Affiliations:** 1 International Crops Research Institute for the Semi-Arid Tropics (ICRISAT), Patancheru, Andhra Pradesh, 502 324, India

## Abstract

**Background:**

Pearl millet [*Pennisetum glaucum *(L.) R. Br.] is a staple food and fodder crop of marginal agricultural lands of sub-Saharan Africa and the Indian subcontinent. It is also a summer forage crop in the southern USA, Australia and Latin America, and is the preferred mulch in Brazilian no-till soybean production systems. Use of molecular marker technology for pearl millet genetic improvement has been limited. Progress is hampered by insufficient numbers of PCR-compatible co-dominant markers that can be used readily in applied breeding programmes. Therefore, we sought to develop additional SSR markers for the pearl millet research community.

**Results:**

A set of new pearl millet SSR markers were developed using available sequence information from 3520 expressed sequence tags (ESTs). After clustering, unigene sequences (2175 singlets and 317 contigs) were searched for the presence of SSRs. We detected 164 sequences containing SSRs (at least 14 bases in length), with a density of one per 1.75 kb of EST sequence. Di-nucleotide repeats were the most abundant followed by tri-nucleotide repeats. Ninety primer pairs were designed and tested for their ability to detect polymorphism across a panel of 11 pairs of pearl millet mapping population parental lines. Clear amplification products were obtained for 58 primer pairs. Of these, 15 were monomorphic across the panel. A subset of 21 polymorphic EST-SSRs and 6 recently developed genomic SSR markers were mapped using existing mapping populations. Linkage map positions of these EST-SSR were compared by homology search with mapped rice genomic sequences on the basis of pearl millet-rice synteny. Most new EST-SSR markers mapped to distal regions of linkage groups, often to previous gaps in these linkage maps. These new EST-SSRs are now are used by ICRISAT in pearl millet diversity assessment and marker-aided breeding programs.

**Conclusion:**

This study has demonstrated the potential of EST-derived SSR primer pairs in pearl millet. As reported for other crops, EST-derived SSRs provide a cost-saving marker development option in pearl millet. Resources developed in this study have added a sizeable number of useful SSRs to the existing repertoire of circa 100 genomic SSRs that were previously available to pearl millet researchers.

## Background

Pearl millet [*Pennisetum glaucum *(L.) R. Br.] is the staple food and fodder crop of millions of poor people living on the most marginal agricultural lands of sub-Saharan Africa and the Indian subcontinent. Indeed, in some of the hottest and driest regions where agriculture is possible in India and Africa, pearl millet is the only cereal that can be grown under dryland conditions and so plays a critical role in food security. However, people living in these regions have not yet benefited much from the current 'biotechnology revolution', or even the 'green revolution' that dramatically increased food grain production on irrigated lands over a generation ago. In recent times, the use of molecular marker technology for the genetic improvement of pearl millet has made some headway, and pearl millet has been elevated to the status of a molecular crop through a series of collaborative projects involving the John Innes Centre (JIC), ICRISAT and their partners supported by the Plant Sciences Research Programme of the UK's Department for International Development (DFID) [1], and the Generation Challenge Programme (GCP) of the Consultative Group on International Agricultural Research (CGIAR).

The first major milestone was achieved in 1993 with the creation of a molecular marker-based genetic linkage map of the pearl millet genome with 181 restriction fragment length polymorphism (RFLP) markers – the marker system of choice in the early 1990s [2]. Now more than 600 molecular markers have been created and mapped for pearl millet, a consensus linkage map has been produced [3], and quantitative trait loci (QTL) for disease resistance [4-8], drought tolerance [9-11], flowering time and grain and stover yield [12], and ruminant nutritional quality of straw [13] have been mapped. These genetic tools for marker-assisted breeding of pearl millet are now in place and available for anyone to use in improving pearl millet hybrids and to extend the economic lifespan of elite hybrid parental lines. However, application of these discoveries is hampered by the limited availability of repeatable, polymorphic PCR-compatible markers for pearl millet. ICRISAT and its partners have successfully demonstrated the use of RFLP markers in the marker-assisted backcross transfer of additional downy mildew resistance into a parental line of popular pearl millet hybrid "HHB 67" and a new version of this hybrid, "HHB 67 Improved" based on the improved parental line, was released for commercial cultivation in 2005 as the first public-bred product of marker-assisted breeding in India [14,15]. However, RFLP markers are too labor-intensive and high cost for applied use, as well as having potential health and environmental hazards. Thus these markers are not considered suitable for large-scale genotyping applications in an applied plant breeding program.

For regular plant breeding applications, PCR-compatible markers based on microsatellites or simple sequence repeats (SSRs) are often considered the most appropriate. SSRs typically provide single-locus markers, which are often co-dominantly inherited and characterized by hypervariability, abundance and reproducibility. Currently, circa 110 SSR markers are available for pearl millet in public domain out of which 65 SSR loci have been mapped [16-20], but a much larger number is required for their application in plant breeding. Therefore, development of additional SSR markers is a valuable objective for the pearl millet research community. In the past, SSRs have been expensive to develop and this has largely limited their application to the more commercially important crops. Enrichment protocols have been used to reduce these costs by focusing sequencing efforts on DNA clones that are likely to contain a particular repeat motif as done by Budak and co-workers. [17].

Recent studies have indicated large numbers of SSRs in coding regions [21,22], which can act as an alternative source of microsatellites. Discovery of microsatellites in Expressed Sequence Tags (ESTs) provides the opportunity to develop SSR markers (EST-SSRs) in a simple and direct way, *i*.*e*., by electronic searches (data mining) of EST databases. Exploitation of this source of SSR markers is obviously limited to the species for which EST sequence information is available. In recent times, lots of efforts have gone into generation of substantial expressed sequence tag (EST) databases for plant species. The exploitation of EST databases to develop microsatellite markers was first attempted in rice [23] and has subsequently been reported from many other plant species [24].

EST-SSRs constitute a novel source of markers that are physically associated with coding regions of the genome, and this can enhance the role of genetic markers in germplasm evaluation by enabling assay of the variation in transcribed sequences and genes where function is known. Contrary to EST-SSRs, genomic SSRs are anonymous markers, and the genetic linkage they sometimes exhibit with quantitative trait loci is often only due to a physical linkage and statistical relationship [25]. In addition, EST-SSRs are easily transferable to closely related species [26,27]. As EST-SSR markers target coding regions of the genome, they facilitate the use of the candidate gene mapping approach, thereby facilitating the dissection of complex traits [28]. This marker system offers the possibility of selecting markers according to the biochemical and physiological properties of their gene products in relation to the phenotype [29]. Considering the advantages and utility of EST-SSR markers, we attempted to develop them from the limited number of EST sequences available for pearl millet.

## Results & discussion

### Identification of SSR

The total number of EST sequences available for pearl millet was 3520 at the start of this study. These sequences spanning the length of 1.4 Mb was assembled using CAP3. The CAP3 analysis resulted in identification of 2175 singlets and 317 contigs. The singlets and contig sequences (1.1 Mb) were searched for SSR motifs. There were 164 sequences containing SSRs, out of which 28 had more than one SSR. The total number of SSR motifs identified was 199 including 23 SSRs in compound form. Thus the frequency of SSRs was 6.6 per cent and comparable with other cereals [30] and the density was one SSR in 1.75 kb of EST, which is comparable to sorghum, rice [31] and wheat [21]. However, in the study of Varshney *et al*. [30] SSRs were reported to occur at a frequency of every 4–8 kb in cereals (barley, maize, rice, rye, sorghum and wheat). Varied results could be found in terms of frequency of SSRs in literature because the parameters used for identification of SSRs as well as the number of sequences assayed varied across studies. SSRs have been found in a range of 6–11% of the total EST sequences from several cereal species *viz*., barley, maize, rye, rice, sorghum and wheat [30]. However, Kantety and co-workers reported comparatively low frequencies of SSR motifs (1.5% in maize to 4.7% in rice) in the EST collections of cereals as they considered SSRs of only 18–20 nucleotides in length [31]. It should be noted that the frequency and density estimates in this study might not reflect the exact picture for pearl millet considering the limited number of EST sequences analysed.

Unlike other crops, di-nucleotide repeats were the most abundant group detected in this pearl millet study. When SSRs of 12 bp in length were considered, tri-nucleotide repeats were more frequent than di-nucleotide repeats in other cereals [31]. In the current study, the relative abundance of di-, tri-, tetra-, penta and hexa-nucleotide repeat motifs was 81 (40%), 53 (27%), 24 (12%), 29 (15%) and 12 (6%), respectively. Among the di-nucleotide motifs, AG/CT was the most abundant (81.5%), which was in line with other cereals [31]. The AG/CT motif can represent codons GAG, AGA, UCU, and CUC in mRNA populations and translate into the amino acids Arg, Glu, Ala, and Leu, respectively. Ala and Leu are present in high frequencies in proteins and this could be one of the reasons for the predominance of AG/CT motifs in EST collections [31]. The least frequent di-nucleotide repeat class was CG (1.23%). In other studies also, CG was found to be the least common motif in most of the cereal species except barley [30,32]. Although AT motifs are reported to be dominant in plant genomes [33-35]; this motif was present only at the rate of 6.17% in the present study. Among the tri-nucleotide repeats, AGC/CGT was the single largest repeat class (26.42%).

Out of 199 SSRs, 141 were Type II microsatellites (having less than 20 bases). In general, it has been observed that repeat numbers and total lengths of SSRs in transcribed regions are relatively small compared to other regions of genome [31,36].

### SSR amplification

Primer pairs could not be synthesized for about 40% of the SSR-containing EST sequences, as the microsatellite was located close to one of the ends of the sequence or flanking sequences were inappropriate for designing high quality primer pairs. Thus, primer pairs were synthesized from 90 unigene sequences only. These primer pairs were used to amplify genomic DNA of 11 pairs of pearl millet mapping population parental lines. Out of 90 primer pairs developed, 15 did not work, 17 produced multiple or non-specific fragments and 58 were functional giving simple PCR products. Most of the primer pairs that failed and the primer pairs that amplified non-scorable products had been designed from EST sequences with compound SSRs or from contig sequences.

Thirty-five percent of the primer pairs designed for pearl millet EST-SSRs did not amplify a product or give simple PCR products. However, a larger percentage of genomic SSR primer pairs have reportedly failed to amplify in pearl millet (55%) [16]. The lack of amplification by some of the EST-SSR markers has been routinely reported in several previous studies in other crops [36-40], which were attributed to primer mismatches, the extension of primers across a splice site or the presence of large introns in the genomic DNA template. The quality of EST sequence is therefore crucial if one is to obtain functional primers with good amplification profiles. Nevertheless the success rate is higher with EST-SSRs than with genomic SSRs. In maize, it has been noted that a higher percentage of primer pairs from enriched genomic libraries failed to give consistent amplification products than from EST-sequence-derived primer pairs in maize [41]. The authors commented that this could be due to SSR candidates being located in repeated or complex sequences in the genomic clones as opposed to lower-copy sequence origins for the EST-derived candidates. In addition, redundancy of clones within the enriched genomic libraries limited the efficiency of identifying novel SSRs.

Non-specific amplifications were a problem for 18% of the primer pairs that we analysed. These primer pairs produced more bands than expected. A similar trend has also been observed in tall fescue and this might be due to amplifications of loci from the duplicated genomic regions [39]. In wheat also, 39% of EST-SSR markers detected multiple loci [38], which is probably due to the high rate of conservation of EST-SSRs across the three genomes of this species, as well as 25–30% gene duplication [42].

There were some differences observed between the expected and the observed sizes of the amplification products, which has been a regular phenomenon with EST-SSRs [43,26]. Among 58 primer pairs producing good quality amplification product, 9 returned product sizes larger than expected, which could be due to simultaneous amplification of an intron during the PCR. Conversely, 9 primer pairs gave amplification products that were smaller than expected, suggesting the possibility of deletions within the genomic sequences or lack of specificity of some primer pairs, which might have resulted in the amplification of a different copy belonging to the same multi-gene family. In a few cases, the presence of introns and insertions-deletions (in-dels) has been confirmed by sequence analysis [39]. The forward and reverse sequences, annealing temperatures, and expected product sizes for the 58 functional primer pairs are provided (see Additional file 1).

### Assessment of polymorphism in mapping population parental pairs

Out of 58 functional EST-SSR primer pairs, 15 were found monomorphic across the surveyed panel of mapping population parental lines. The average number of alleles detected across the surveyed panel of genotypes for the 43 polymorphic loci was just 3.1. Nineteen primer pairs produced only 2 alleles across this panel. The highest number of alleles (8) was produced by primer pair ICMP3008. As expected, the genomic SSRs tested were comparatively more polymorphic. Out of 18 genomic SSR primer pairs (CTM series), 4 did not amplify and for the remaining 14 functional primer pairs, 10 detected polymorphism in the surveyed panel of parental genotypes. The average number of alleles produced by these 10 genomic SSRs was 5.0. The polymorphism information for EST-SSRs and genomic SSRs across the set of mapping population parental lines are provided (see Additional file 2 and 3).

It has been reported that EST-derived SSRs are less polymorphic than those derived from genomic libraries. However, EST-SSRs represent a unique opportunity to exploit existing sequence information and by-pass creation and sequencing of SSR-enriched (or random) genomic libraries. Perhaps the most important feature of EST-SSRs is that the primer pairs designed for them are more likely to function in distantly related species than are SSR primer pairs derived from genomic libraries [31,44]. This makes EST-SSRs potentially more useful for comparative mapping studies. Once mapped, EST-SSRs provide map locations for the genes that carry them. We therefore propose to use these markers in other less studied crops like finger millet (*Eleusine coracana*) and foxtail millet (*Setaria italica*), and have initiated assessment of the ability of the pearl millet EST-SSR primer pairs described here to amplify product and detect polymorphism in these small millets.

### Mapping of SSR markers

The segregation pattern of loci detected by 17 EST-SSR and 6 genomic SSR primer pairs that were polymorphic between ICMB 841-P3 and 863B-P2 were assessed using 149 F_2 _individuals. One of the genomic SSRs, *Xctm*08 detected by primer pair CTM08, behaved in dominant fashion and segregated in the ratio of 3:1. The remaining 22 markers segregated in a co-dominant manner. However, 11 of the 17 EST-SSR markers did not segregate in the expected ratio of 1:2:1. Instead, they exhibited skewed distributions towards one parental allele or the other. Such segregation distortion has often been noted in pearl millet. Most of such distortions are cross-specific except that distortion on Linkage Group (LG) 4 was present in three of the four crosses analyzed in an earlier study [16]. In all the crosses of that study, there was an excess of one of the parental alleles in the region spanned by markers *Xpsm*265 and *Xpsm*364, suggesting presence of a gene in this region that affects gametophytic or zygotic viability. Previously, segregation distortion was noted on LG 3 and LG 6 of the ICMB 841-P3 × 863B-P2 cross [10,11] with an excess of ICMB 841 alleles. In this study too, 6 out of 11 distorted markers were from LG 6. However the skewness was not only always towards the alleles of female parent ICMB 841, but in some cases favored those of male parent 863B-P2. Out of 6 genomic SSRs, the segregation pattern for one that mapped to LG 3 was distorted.

**Figure 1:**
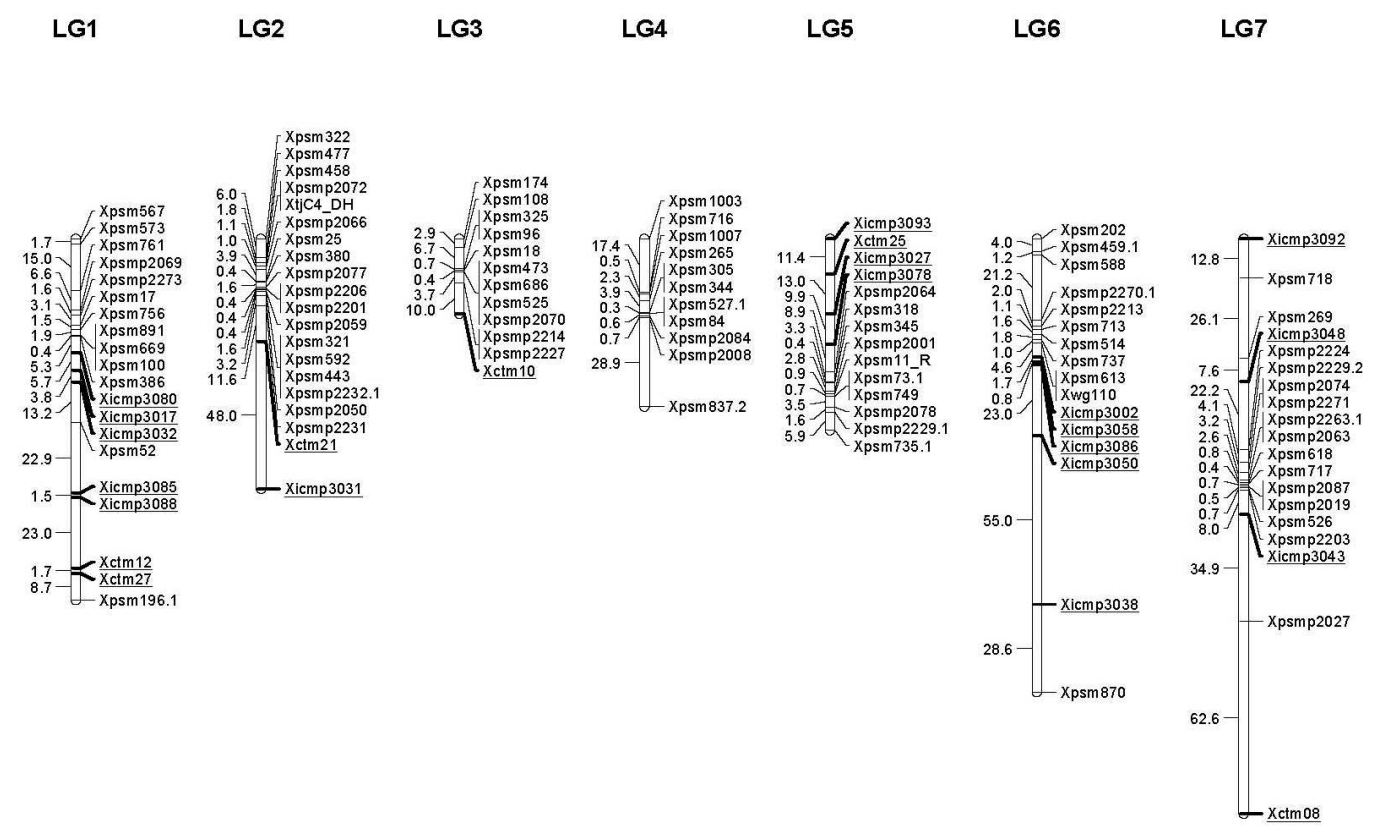
**Linkage map of ICMB 841-P3 × 863B-P2 showing the map positions of new EST-SSR and genomic SSR markers relative to markers previously mapped in this population**. Newly added markers are underlined.

The 17 EST-SSR markers were mapped to 5 of the 7 pearl millet LGs. The map locations of these 17 new EST-SSRs and 6 genomic SSRs are shown in Figure 1. The characteristic feature of pearl millet linkage maps is the presence of large gaps in distal regions of the chromosomes. Most of the RFLP and SSR markers developed to date have mapped to centromeric regions. Past attempts to develop more markers targeting more distal regions of the pearl millet chromosomes have resulted in identification of few additional markers [16]. The EST-SSRs developed in this study were expected to map to gene-rich portions of the genome, which are expected to be associated with non-centromeric regions of the seven pearl millet chromosome pairs.

The previously constructed framework map for the cross ICMB 841-P3 × 863B-P2 [10,11] had large gaps in LG 1 (between *Xpsm*52 and *Xpsm*196.1), LG 2 (between *Xpsm*708.1 and *Xpsm*322; and between *Xpsm*2237 and *Xpsm*708.2), LG 6 (between *Xwg*110 and *Xpsm*870), and LG 7 (between *Xpsm*718 and *Xpsm*269; and between *Xpsmp*2203 and *Xpsmp*2027). Interestingly, some of the new EST-SSR markers mapped to these gaps. For example, the 63 cM gap between *Xpsm*52 and *Xpsm*196.1 on LG 1 was covered by *Xicmp*3085, *Xicmp*3088, *Xctm*112 and *Xctm*27.

So far, the shortest linkage group in pearl millet was LG 3. This linkage group was thought to be reasonably complete using comparative mapping as a guide [45]. In this study, one additional marker *Xctm*12 was mapped at distal end of LG 3, increasing map length of this LG by 8 cM. The completeness of another short linkage group, LG 5, was not confirmed earlier. In this study, 4 additional markers (*Xicmp*3093, *Xctm2*5, *Xicmp*3027, and *Xicmp*3078) were mapped to the top of LG 5 increasing the map length of this LG in this cross to 62 cM from 23 cM. This is in agreement with the earlier prediction that markers would eventually be found that will map 30 cM or more beyond the most distal markers on LG 3 and LG 5 [16,45]. The gap between *Xwg*110 and *Xpsm*870 in LG 6 was filled with 5 new markers but the distance between these two markers increased drastically, calling into question the positioning of these markers.

The average map distance between new markers mapping to the ends of the LGs was comparatively high in this study. This supports the hypothesis of high recombination rates in distal regions of pearl millet chromosome postulated by previous studies [2,16,45]. Gene-rich regions are usually subject to higher recombination rates compared to gene-free regions, which are mainly composed of repetitive DNA [46]. Four additional EST-SSR loci (*viz*., *Xicmp*3024, *Xicmp*3025, *Xicmp*3045 and *Xicmp*3066), which were monomorphic in the ICMB 841-P3 × 863B-P2 cross, were mapped using the F_2 _population of the cross 81B-P8 × IPC 804 (data not shown). *Xicmp*3024 was mapped to LG 6, *Xicmp*3025 to LG 3, *Xicmp*3045 to LG 2 and *Xicmp*3066 to LG 7.

### Comparative Genomics

To confirm map locations of the new EST-SSR markers, the mapped EST microsatellite sequences were aligned against the rice genome. While six of the mapped pearl millet EST-SSR sequences found no hits on the rice genome, the remaining EST sequences had one or more rice homologues that were physically mapped. There was correspondence between pearl millet linkage groups and rice chromosomes as expected from rice-pearl millet synteny [45]. The homologous rice sequences for each pearl millet EST sequence corresponding to a mapped SSR, and their putative functions, are given (see Additional file 4).

The integration of RFLP markers previously mapped in other grass species has provided the anchor points to align the pearl millet linkage groups to other cereal genetic maps. The complex relationship of pearl millet with foxtail millet and rice has been studied using RFLP probes [45]. So far most of the comparative genomics efforts have relied heavily on the hybridization-based RFLP technique with lower resolution of microsynteny [47]. However, recently, sequence-based comparative maps have been developed for rice-wheat [48] and sorghum-rice [49] that enhance the resolution considerably. The application of a PCR-based co-dominant marker system for comparative genomics would be highly desirable, because such a marker system can increase the efficiency of transferring genetic information across species. Due to their transferability, EST-SSR markers have good potential for such applications in comparative genomics [31,36,50]. Thus the EST-SSR developed in this study offer great potential in comparative genomics studies in pearl millet with other cereals.

## Conclusion

The frequency of SSR-containing ESTs was quite high in this initial sample of the expressed portion of the pearl millet genome, so it appears that this approach can add reasonable numbers of SSRs to the existing pearl millet SSR collection at very modest cost provided that 1) the sequence information is available freely (or at very low cost) as a result of other research programs, 2) care is taken to minimize redundancy, and 3) primer synthesis and testing is limited to only sequences flanking the most highly repeated di-, tri-, and tetra-nucleotide motifs. This study has demonstrated the potential utility of EST-derived SSR primers in pearl millet. As reported for other crops, EST-derived SSRs provide a cost-saving marker development option in pearl millet. These resources will add a sizeable number of relatively more useful SSRs to the existing repertoire of circa 100 genomic SSRs that are already available to pearl millet researchers. These new SSR markers are being used in our on-going marker-aided backcross program for improvement of downy mildew resistance, drought tolerance and stover yield and quality traits in pearl millet.

## Methods

### Identification of SSR and primer designing

In this study, 3520 pearl millet EST sequences (1.46 Mb) obtained from cDNA libraries constructed from root and shoot tissues of seedlings exposed to cold, drought and salinity stresses were used. These EST sequences were made available by our collaborators in International Center for Genetic Engineering and Biotechnology (ICGEB), New Delhi and University of Hyderabad, Hyderabad, India. Out of 3520 sequences, 2810 are currently available in the National Center for Biotechnology Information (NCBI) GenBank database (accession numbers 32275159–32277652 and 91982272–91982588). These EST sequences were assembled using CAP3 [51] to identify unigenes. The unigene sequences (singlets and contigs) were then searched for the presence of SSR motifs using the MIcro SAtellite identification tool (MISA) available at . The search was restricted to motifs having at least 14 bp length (*i*.*e*., di-nucleotide > 7; tri-nucleotide > 5; tetra-nucleotide > 4; penta-nucleotide > 3 and hexa-nucleotide > 3) and the minimum difference between two SSRs in a sequence (interruption) was 100 bases. From the SSR-containing EST sequences, primer pairs were designed targeting the SSRs except hexa-nucleotide repeats using Primer3 [52]. The primer designing conditions were: 57–61°C melting temperature, 40–60% GC content, and 18–24 bp primer length.

### Plant materials

The primer pairs developed from SSR-containing ESTs (prefixed with 'ICMP' for ICRISAT Millet Primer), hereafter referred to as 'EST-SSRs' along with a set of 18 SSR primer pairs (prefixed with 'CTM'), hereafter referred to as 'genomic SSRs' developed from small insert genomic library of pearl millet [18] were tested for their ability to detect polymorphism among 11 pairs of pearl millet mapping population parental lines available at ICRISAT-Patancheru *viz*., PRLT 2/89-33 and H 77/833-2; ICMB 841-P3 and 863B-P2; Tift 23D_2_B_1_-P5 and WSIL-P8; PT 732B-P2 and P 1449-P1; LGD 1-B-10 and ICMP 85410-P7; 81B-P6 and ICMP 451-P8; ICMP 451-P6 and H 77/833-2-P5(NT); W 504-1-P1 and P 310-17; IP 18293-P152 and Tift 238D_1_-P158; ICMB 89111-P6 and ICMB 90111-P6; 81B-P8 and IPC 804. The origin and characteristics of these 22 inbred lines are given (see Additional file 5).

Two mapping populations were used to map the polymorphic markers. The first population consists of 149 F_2 _individuals of the cross ICMB 841-P3 × 863B-P2, which has been previously used for mapping drought tolerance, downy mildew resistance, and stover quality QTLs [10,11,13]. The other population consists of 397 F_2 _individuals of the cross 81B-P8 × IPC 804, which was originally developed to map fertility restorer genes [53].

### DNA extraction

Genomic DNA was extracted from leaf tissue of F_3 _bulks representing each of the individual entries from the segregating F_2 _population as described by Sharp *et al*. [54]. The DNA concentrations were determined by comparing the sample intensity with that of known amounts of uncut lambda DNA by electrophoresis in 1.2% agarose gels containing ethidium bromide. DNA was diluted to a working stock concentration of approximately 5 ng/μl and checked before use.

### PCR and Electrophoresis

PCRs were carried out in a 10 μl reaction mixture containing 10–15 g of genomic DNA, 2 pmol of each primer, 1 mM MgCl_2_, 0.1 mM of each dNTP, 1× reaction buffer, and 0.2 U *Taq *polymerase (Bioline). After one denaturing step of 3 min at 94°C, a touchdown amplification program was performed on GeneAmp 9700 thermal cycler (Applied Biosystems, USA). This profile consisted of a denaturing step of 25 sec at 94°C and an extension step of 30 sec at 72°C. The initial annealing step was 20 sec at 64°C for one cycle and subsequently the temperature was reduced by 1°C for every cycle until a final temperature of 55°C was reached. The annealing temperature of 55°C was maintained for the last 35 cycles of amplification, followed by final extension of 72°C for 7 min. PCR products were size-separated on native polyacrylamide gels (6%) run on 0.5× TBE buffer at 600 V for 3 hours using a Bio-Rad^® ^sequencing gel apparatus. After electrophoresis, the banding patterns of PCR product on PAGE gels were visualized by silver-staining [55].

### Polymorphism assessment and mapping of EST-SSRs

The new markers were placed onto the already available framework linkage maps of the crosses ICMB 841-P3 × 863B-P2 with 55 RFLP and 32 SSR markers [10,11] and 81B-P8 × IPC 804 with 11 RFLP, 23 SSR and 3 morphological markers (53), using Mapmaker 3.0 [56] at LOD 4. The 'build' command with the LOD of 4 was used to incorporate SSR markers into the frameworks. Local orders were verified with the 'ripple' command (window size of 5 loci, LOD 3). Map distances were calculated based on the Haldane mapping function. The mapped EST-SSRs were aligned against the rice genome using the BLAST (WU-BLAST 2.0) against the IRGSP pseudomolecules Build04 . Only those sequences from rice that returned an e-value of e-10 or less during the BLAST search were considered putative homologs. BLAST searches were carried out in June 2006 and again validated in April 2008.

## Authors' contributions

SS designed the study, analysed the data and drafted the manuscript. BJ and VM were involved with *in-silico *analysis. PSK and SN performed initial optimization of primers and a part of wet lab experiments. TN was partly involved in data generation and analysis. CTH provided overall guidance in designing and performing this study and edited the manuscript. All the authors read and approved the manuscript.

## Supplementary Material

Additional file 1 **Details of newly synthesized EST-SSR primer pairs.** Click here for file

Additional file 2 **Polymorphism information of EST-SSRs on parental lines of pearl millet mapping populations.** Click here for file

Additional file 3 **Polymorphism information of genomic SSRs on parental lines of pearl millet mapping populations.** Click here for file

Additional file 4 **Positions of rice homologs of mapped ESTs and their putative functions.** Click here for file

Additional file 5 **Details of pearl millet inbred lines used for polymorphism testing.** Click here for file
