# Supplementary material for: Development and mapping of Simple Sequence Repeat markers for pearl millet from data mining of Expressed Sequence Tags
Source: BMC Plant Biol. 2008 Nov 27;8:119. doi: 10.1186/1471-2229-8-119 (PMC2632669; doi:10.1186/1471-2229-8-119)
Supplement: Additional file 1 — Details of newly synthesized EST-SSR primer pairs. [file 1471-2229-8-119-S1.doc]

Additional file 1. Details of newly synthesized EST-SSR primer pairs

| S. No. | Primer pair | Genbank EST ID^1^ | SSR motif | Forward sequence (5’-3’) | Reverse sequence (5’-3’) | Tm (°C)^2^ | | PIC |
| --- | --- | --- | --- | --- | --- | --- | --- | --- |
|  |  |  |  |  |  | F | R |  |
| 01 | ICMP3001 | CD725965 | (AAAC)4 | ACATGGAGTTGGCACCAGAT | GGAATGAAAGGAAGCCAACA | 60.0 | 60.1 | 0.290 |
| 02 | ICMP3002 | CD724823 | (AAG)7 | AAGATGGATGATGGATTGATGA | TACACACACATTGCCACACG | 60.4 | 60.0 | 0.370 |
| 03 | ICMP3004 | CD724688 | (AC)14 | TGTTACGCAGTGCTCGGTAG | ATATAGGGGCGCGCAATAGT | 60.1 | 60.8 | 0.480 |
| 04 | ICMP3005 | CD725868 | (CA)14 | CGCGGTGTTCTCACACAC | TGTGAATTCCGCGGGTATAG | 59.9 | 60.9 | 0.208 |
| 05 | ICMP3006 | CD726292 | (AC)16 | AAATCGGTCGTGGTGAAGTT | GAGAATGTGGGAGACACACG | 59.5 | 59.1 | 0.000 |
| 06 | ICMP3008 | CD726692 | (AC)18 | GCACGAGGGTTGATTAGGC | CTCAATAAGAGGGGCGAGAA | 60.6 | 59.4 | 0.000 |
| 07 | ICMP3010 | CD726627 | (AC)19 | TGTCTCGAGAGCAGGTGATG | AGAATGTGGGGGAGACACAC | 60.1 | 59.8 | 0.000 |
| 08 | ICMP3013 | CD726663 | (AC)33 | TGTGGGAGAGAGGAGAGTCC | ACATGGAGTTGGCACCAGAT | 59.4 | 59.2 | 0.400 |
| 09 | ICMP3014 | CD724961 | (ACC)8 | GCTATTGCCACTGCTTCACA | AACACCCACCATGCAACAG | 60.0 | 60.4 | 0.083 |
| 10 | ICMP3016 | CD724583 | (CA)17 | GTCAACCATTTGGGCTCACT | GTCAACCATTTGGGCTCACT | 60.0 | 61.4 | 0.000 |
| 11 | ICMP3017 | CD724750 | (CAG)7 | CACCAAACAGCATCAAGCAG | AGGTAGCCGAGGAAGGTGAG | 60.5 | 60.8 | 0.508 |
| 12 | ICMP3018 | CD724312 | (CATG)4 | ACGAGGACAAGCTCTTGGAA | ACGGCGCATACTCGATCATA | 60.0 | 61.2 | 0.000 |
| 13 | ICMP3024 | CD724892 | (GCCGT)4(CT)11 | ATCGAGGCCAAGTACGTGAT | CGAGCTTCTAGCTCCAATCC | 59.6 | 59.2 | 0.638 |
| 14 | ICMP3025 | CD724352 | (CTC)6 | GTTGCAGATGAGCGATCGTA | CGCCGACCAAGAACTTCATA | 60.0 | 61.2 | 0.083 |
| 15 | ICMP3027 | CD724428 | (GAT)6 | GTTTCTCCGGGCATGTGTTA | AGTGACCTGGGGTACAGACG | 60.9 | 60.0 | 0.655 |
| 16 | ICMP3028 | CD724780 | (GATC)4 | ACGATTCTTCGTCGTTCCAG | GATACGCGCGAGCTACATTT | 60.3 | 60.4 | 0.083 |
| 17 | ICMP3029 | CD726384 | (GCA)6(GCA)5 | ATCGATCTGTTCCACCCAGT | GGACTGGTACTGCTGCTGCT | 59.4 | 60.6 | 0.297 |
| 18 | ICMP3031 | CD725489 | (GCG)6 | CACGCTGCTGGAACTTATCA | TCTCTCTCTCGGATCGCTGT | 60.0 | 60.3 | 0.000 |
| 19 | ICMP3032 | CD725922 | (GCT)8 | GCGTAGACGGCGTAGATGAT | CAACAGCATCAAGCAGGAGA | 60.3 | 60.1 | 0.516 |
| 20 | ICMP3037 | CD725608 | (TGGA)4 | CGCTGCGTTTATTGAAGGAG | GGCGAAACAAAGAGAGTTGG | 60.9 | 59.9 | 0.083 |
| 21 | ICMP3038 | CD724894 | (TGT)6 | CTCTCGGTTTGACGGTTTGT | GAGGAGCAGGATACCCCTTT | 60.2 | 59.5 | 0.083 |
| 22 | ICMP3039 | CD726152 | (TGT)6 | GGCACGAGGGGCTAAGTAAG | GCCCTCCGTCGTCATTACAT | 60.1 | 62.2 | 0.558 |
| 23 | ICMP3042 | CD724326 | (GT)7 | TAGTTAATGGGGGTGCGTGT | AAGCACCATCAGCATACCC | 60.3 | 58.6 | 0.099 |
| 24 | ICMP3043 | CD724407 | (AGC)5 | TCCTGTACAAGGACGTGCAG | TATCGACGCCAACGATACTG | 59.9 | 59.7 | 0.319 |
| 25 | ICMP3045 | CD724462 | (AAG)5(CAG)5 | ACAAGGACGACAAGGACCAC | CCTCTCCAAGCACATGTTTC | 60.0 | 58.3 | 0.152 |
| 26 | ICMP3047 | CD724492 | (GT)7 | CGGAGACGCACTAGACTTGG | ACCACCATTCCATCACTCCT | 61.0 | 59.2 | 0.000 |
| 27 | ICMP3048 | CD724492 | (GTGCG)3 | CGGAACTGCTGGAGTGAAAT | GCGACTTCGACCGACTTTT | 60.3 | 60.4 | 0.091 |
| 28 | ICMP3049 | CD724555 | (CCTG)4(TGCCC)3 | GAGCTGAACACGCTCAAGG | CAGATGACATCCATCCGTTG | 59.7 | 59.9 | 0.083 |
| 29 | ICMP3050 | CD724749 | (TA)8 | ATGTCCAGTGTTGACGGTGA | CGGGGAAGAGACAGGCTACT | 60.0 | 60.8 | 0.544 |
| 30 | ICMP3051 | CD724943 | (CTAGA)3 | TCTTCTTCCGCATCCTCTGT | GTACCGCCCTTTGTGTTGAT | 60.0 | 59.9 | 0.000 |
| 31 | ICMP3055 | CD725161 | (CGG)5 | CCCAAACGCAAGTAGGGTTA | CCTTCTCCTGCCCCAGAC | 60.0 | 60.8 | 0.000 |
| 32 | ICMP3056 | CD725173 | (TGG)5 | ACGGAGCTACGGTTGGAATA | CACAAGGGACCCCACGATA | 59.6 | 61.8 | 0.152 |
| 33 | ICMP3057 | CD725183 | (GAC)5 | ATGTGGAATAACCGCAGAGG | AGCAAAAGCTGAGCGACTTC | 60.0 | 60.0 | 0.332 |
| 34 | ICMP3058 | CD725199 | (GA)9 | CGGAGCTCCTATCATTCCAA | GCAAGCCACAAGCCTATCTC | 60.2 | 60.0 | 0.158 |
| 35 | ICMP3063 | CD725658 | (GTG)5 | TCCGGTAGAGACCGTAATGG | GGCACTCCCTAGCAAAATGA | 60.0 | 60.2 | 0.558 |
| 36 | ICMP3066 | CD726121 | (AG)7 | GGCCCCAAGTAACTTCCCTA | TGTCAGACACAGATGCCACA | 60.3 | 59.9 | 0.576 |
| 37 | ICMP3068 | CD726394 | (GCT)5 | CTGGCAAAGTTGTAGCGTGA | ATGTCGCTCTCTGCCAAGAT | 60.1 | 60.0 | 0.000 |
| 38 | ICMP3069 | CD726403 | (ATCC)4 | TAGGAGGGGACTGCTCCTTT | AGGAAGAGGATGGTGGTGTG | 60.2 | 60.0 | 0.000 |
| 39 | ICMP3072 | CD726578 | (CGC)5 | CGCAGCTCTACTTCCAGACC | CAGTAACCAAGAGCACCTCGAT | 60.2 | 60.7 | 0.000 |
| 40 | ICMP3076 | CD725560 | (CGCAT)3 | CACGAGGCAGAAGCACATT | CTCTTCTCGGCGATGAGC | 60.0 | 59.8 | 0.000 |
| 41 | ICMP3077 | CD726162 | (CCGGG)3 | AGCATCCCCTACACCATCAG | CTCTTCTCTCGCACACATGC | 60.0 | 59.7 | 0.292 |
| 42 | ICMP3078 | CD726702 | (TGCCA)3 | TCCAGACAGTTCAGCAGGTG | CCACACGAGACAGAGCACAC | 60.0 | 60.1 | 0.305 |
| 43 | ICMP3079 | CD726782 | (CTTTT)3 | ATGGTAGAGCGGTGAGGTTG | GCAAGGCAATGTAGGTGGAT | 60.1 | 60.0 | 0.339 |
| 44 | ICMP3080 | CD724750 | (AGC)8 | CAAACAGCATCAAGCAGGAG | GCGTAGACGGCGTAGATGAT | 59.6 | 60.3 | 0.649 |
| 45 | ICMP3081 | EB411027 | (CTA)5 | ACGCCGTTTTCGTGTAGTCT | TCCACAAGGTGACCTCACTG | 59.8 | 59.7 | 0.597 |
| 46 | ICMP3085 | EB411016 | (TCA)5 | CTGAAGCTGAAGAGGCCTTG | GGCGGAGATCAGAGTTCG | 60.3 | 59.5 | 0.466 |
| 47 | ICMP3086 | EB410970 | (CAT)5 | ACCAAACGTCCAAACCAGAG | ATATCTCTTCGCTGCGGTGT | 60.0 | 59.9 | 0.455 |
| 48 | ICMP3088 | EB411043 | (TCC)8(TCTA)4 | TCAGGTGGAGATCGATGTTG | TTACGGGAGGATGAGGATG | 59.6 | 59.9 | 0.813 |
| 49 | ICMP3091 | NA | (AGG)5 | AACAAGGACCTGCGATTCAC | CATGACAGCAACGACGAATC | 60.1 | 60.3 | 0.158 |
| 50 | ICMP3092 | NA | (TAG)5 | GTTGCTGTCATGTCGTCTGG | CATCATGCCTGTGAGCAATG | 60.2 | 61.3 | 0.579 |
| 51 | ICMP3093 | NA | (AGC)5 | AGTTTCCAATCCCACCCTCT | GTTGGAGATGAGGTCGAGGT | 59.8 | 59.1 | 0.562 |
| 52 | ICMP3094 | NA | (AAC)5 | GACCTCGACCTCATCTCCAA | CGACAGCGAACTGGGATTC | 60.2 | 60.7 | 0.000 |
| 53 | ICMP3095 | NA | (TAGAT)3 | GGGAGGCCACGATTTAAAGA | ACAATGTGCACGCAAGGA | 61.3 | 59.8 | 0.000 |
| 54 | ICMP3096 | NA | (CTT)5 | CTGCATTGCAACATCCTCAC | AACCTGCAGTGGAAGCAATC | 60.3 | 60.3 | 0.269 |
| 55 | ICMP4006 | CD724950 | (TG)7 | TGAGGACCGAGAAGAAGCAT | CAACACCCAACAGAAACTGAA | 58.7 | 59.0 | 0.000 |
| 56 | ICMP4007 | CD725208 | (AGC)5 | ATGTCCATTGCATCTCCGTA | TTGGCGATATCCTAAAAATGG | 59.0 | 59.0 | 0.000 |
| 57 | ICMP4010 | EB411015 | (CCGG)4 | ATCCCCTACAGCATCAGCAC | CGGCGGAGAGATCTTATTCA | 60.1 | 60.3 | 0.362 |
| 58 | ICMP4014 | NA | (ATA)9 | TTCCTTCAATACACAGTTGTTGG | ACCATGAGGACCTTGACCAG | 59.1 | 60.0 | 0.529 |

^1^NA – Not available (contig sequence or not yet submitted to Genbank)

^2^F – Forward primer; R – Reverse primer
