# Supplementary material for: Development and mapping of Simple Sequence Repeat markers for pearl millet from data mining of Expressed Sequence Tags
Source: BMC Plant Biol. 2008 Nov 27;8:119. doi: 10.1186/1471-2229-8-119 (PMC2632669; doi:10.1186/1471-2229-8-119)
Supplement: Additional file 2 — Polymorphism information of EST-SSRs on parental lines of pearl millet mapping populations. [file 1471-2229-8-119-S2.doc]

Additional File 2 Polymorphism information of EST-SSR on parental lines of pearl millet mapping populations

| Primer pair | Approximate product size (bp) | PRLT 2/89-33 | H 77/833-2 | ICMB 841-P3 | 863B-P2 | Tift 23D2B1-P1-P5 | WSIL-P8 | PT 732B-P2 | P1449-2-P1 | LGD 1-B-10 | ICMP 85410-P7 | 81B-P6 | ICMP 451-P8 | ICMP 451-P6 | H 77/833-2-P5(NT) | W 504-1-P1 | P310-17-Bk | IP 18293-P152 | Tift 238D1-P158 | ICMB 90111-P6 | ICMB 89111-P6 | IPC 804 | 81B-P8 |
| --- | --- | --- | --- | --- | --- | --- | --- | --- | --- | --- | --- | --- | --- | --- | --- | --- | --- | --- | --- | --- | --- | --- | --- |
| ICMP3001 | 195-200 | b | b | a | a | a | a | a | a | a | a | a | a | a | b | b | a | a | a | a | b | a | a |
| ICMP3002 | 200-230 | a | b | a | b | a | a | b | b | - | b | b | b | b | b | a | a | a | a | b | a | b | b |
| ICMP3004 | 200-210 | b | - | a | - | - | - | - | a | b | b | b | - | a | b | c | - | - | - | b | a | - | a |
| ICMP3005 | 185-190 | a | a | a | a | a | a | a | a | a | a | a | a | a | a | a | a | a | a | b | b | a | b |
| ICMP3006 | 120 | a | a | a | a | a | a | a | a | a | a | a | a | a | a | a | a | a | a | a | a | a | a |
| ICMP3008 | 310 | a | a | a | a | a | a | a | a | a | a | a | a | a | a | a | a | a | a | a | a | a | a |
| ICMP3010 | 100 | a | a | a | a | a | a | a | a | a | a | a | a | a | a | a | a | a | a | a | a | a | a |
| ICMP3013 | 210-250 | a1 | a1 | a,a1 | a1 | a,a1 | a1 | a,a1 | a1 | a,a1 | a1 | a,a1 | a1 | a1 | a1 | a,a1 | a1,b | a1 | a,a1 | a1 | a,a1 | a1 | a,a1 |
| ICMP3014 | 220-230 | a | a | a | a | a | a | a | a | a | a | a | a | a | a | a | a | a | a | b | a | a | a |
| ICMP3016 | 700 | a | a | a | a | a | a | a | a | a | a | a | a | a | a | a | a | a | a | a | a | a | a |
| ICMP3017 | 180-185 | a | b | a | c | a | a | a | a | b | b | b | a | b | a | b | a | c | c | b | a | a | a |
| ICMP3018 | 210 | a | a | a | a | a | a | a | a | a | a | a | a | a | a | a | a | a | a | a | a | a | a |
| ICMP3024 | 190-200 | a,c | a | a | a | a | a | a | d | c | c | a | a,c | c | d | a,b | d | a | b | d | - | b | c |
| ICMP3025 | 200-205 | a | a | a | a | a | a | a | a | a | a | a | a | a | a | a | a | a | a | a | a | b | a |
| ICMP3027 | 210-220 | a | c | c | b | a | c | a | - | c | a | d | c | c | b | a | c | d | a | b | - | b | - |
| ICMP3028 | 160-165 | a | a | a | a | a | a | a | a | a | a | a | a | a | a | a | a | b | a | a | a | a | a |
| ICMP3029 | 220-230 | a | b | a | a | a | a | a | a | a | a | a | a | a | b | b | a | - | b | b | a | a | a |
| ICMP3032 | 190-200 | b | a | b | a | a | a | a | c | a | a | a | c | c | a | c | c | - | a | a | b | b | a |
| ICMP3037 | 100 | a | a | a | a | a | a | a | b | a | a | a | a | a | a | a | a | a | a | a | a | a | a |
| ICMP3038 | 95 | a | a | b | a | a | a | a | b | a | a | a | a | a | a | a | a | a | a | a | a | a | a |
| ICMP3039 | 790-800 | - | b | a | b | a | b | a | b | b | a | a | c | c | c | a | - | a | c | a | b | - | a |
| ICMP3042 | 580-585 | a | a | a | a | a | a | a | a | - | a | a | a | a | a | a | a | a | a | b | - | - | - |
| ICMP3043 | 200-210 | a | a,a1 | a | b | a,a1 | a | a | a1 | a | a | a | a | a | a | a | a,b | a | a | b | a | a | a |
| ICMP3045 | 310-320 | a | a | a | a | a | a | a | a | a | a | a | a | a | a | a | a | a | a | a | b | a | b |
| ICMP3047 | 180 | a | a | a | a | a | a | a | a | a | a | a | a | a | a | a | a | a | a | a | a | a | a |
| ICMP3048 | 250-260 | a | a | a | b | a | a | a | a | a | a | a | a | a | - | a | a | a | a | a | a | - | a |
| ICMP3049 | 180 | a | a | a | a | a | a | a | a | a | a | a | a | a | a | a | a | a | a | a | a | a | b |
| ICMP3050 | 220-250 | d | c | a | c | a | b | c | c | c | c | c | d | d | c | c | - | c | - | c | b | b | c |
| ICMP3051 | 180-200 | a | a | a | a | a | a | a | a | a | a | a | a | a | a | a | a | a | a | a | a | a | a |
| ICMP3055 | 190 | a | a | a | a | a | a | a | a | a | a | a | a | a | a | a | a | a | a | a | a | a | a |
| ICMP3056 | 160-170 | a | b | a | a | a | a | a | a | a | a | a | a | b | a | a | a | a | a | a | a | a | a |
| ICMP3057 | 750-755 | b | a | - | b | a | a | - | b | a | a | a | a | a | b | a | a | b | a | b | a | a | a |
| ICMP3058 | 165-175 | b | b | a | b | a | b | - | b | b | b | b | b | b | b | b | b | b | b | b | b | b | b |
| ICMP3063 | 170-180 | a,c | a,b | c | b,c | a | b,c | b,c | b,c | c | c | a,b | b | b | a,b | b,c | b,c | c | a,b | b,c | a,b | - | a,b |
| ICMP3066 | 140-200 | a | c | a | a | a | e | a | d | c | b | a | a | a | a | a | a | d | e | d | a | b | a |
| ICMP3068 | 200 | - | a | a | a | a | a | a | a | a | a | a | - | a | a | a | a | a | a | a | a | a | a |
| ICMP3069 | 190 | a | a | a | a | a | a | a | a | a | a | a | a | a | a | a | a | a | a | a | a | a | a |
| ICMP3072 | 210 | a | a | a | a | a | a | a | a | a | a | a | a | a | a | a | a | a | a | a | a | a | a |
| ICMP3076 | 180 | a | a | a | a | a | a | a | a | a | a | a | a | a | a | a | a | a | a | a | a | a | a |
| ICMP3077 | 330-370 | a | a | a | a | a | a | - | a | c | b | a | a | a | a | a | a | a | b | a | b | a | a |
| ICMP3078 | 240-250 | a | a | a | b | a | b | a | a | a | b | a | a | a | - | - | a | a | b | a | b | a | a |
| ICMP3079.1 | 790-800 | a | c | a | a | a | a | a | d | d | a | a | a | a | a | - | b | a | - | a | a | a | - |
| ICMP3079.2 | 205-210 | a | b | a | a | a | b | a | a | a | b | a | a | a | a | b | a | b | a | a | a | a | b |
| ICMP3080 | 500-540 | c | a | b | e | a | a | - | e | a | e | a | e | e | a | e | e | c | a | c | c | c | d |
| ICMP3081 | 180-210 | - | c | b | - | a | - | d | b | b | b | b | c | - | b | a | b | d | b | a | d | - | b |
| ICMP3085 | 190-200 | - | c | c | b | - | c | b | c | c | c | b | a | a | c | c | c | c | c | c | b | b | b |
| ICMP3086 | 140-150 | b | b | a | b | a | b | b | a | b | a | a | c | c | b | b | - | b | b | b | b | c | b |
| ICMP3088 | 130-160 | e | h | a | e | a | a | f | c | a | a | d | e | e | g | d | g | b | c | c | d | - | d |
| ICMP3091.1 | 530-550 | a | a | a | a | a | a | a | b | a | a | a | a | a | a | a | a | b | a | a | a | - | a |
| ICMP3091.2 | 180-185 | c | a | a | a | a | a | a | b | c | a | c | a | a | a | a | a | a | a | a | a | - | c |
| ICMP3092 | 225-230 | d | b | d | c | a | a | d | d | d | b | c | a | a | d | d | d | a | a | d | d | a | d |
| ICMP3093 | 175-190 | d | d | a | b | a | d | d | b | - | c | a | d | d | d | d | a | a | d | d | - | d | c |
| ICMP3094 | 180 | a | a | a | a | a | a | a | a | a | a | a | a | a | a | a | a | a | a | a | a | - | a |
| ICMP3095 | 250 | a | a | a | a | a | a | a | a | a | a | a | a | a | a | a | a | a | a | a | a | - | a |
| ICMP3096 | 210-220 | a | a | - | a | a | c | a | a | a | b | a | a | a | a | a | a | - | b | a | - | - | a |
| ICMP4006 | 280 | a | a | a | a | a | a | a | a | a | a | a | a | a | a | a | a | a | a | a | a | a | a |
| ICMP4007 | 120 | a | a | a | a | a | a | a | a | a | a | a | a | a | a | a | a | a | a | a | a | a | a |
| ICMP4010.1 | 375-380 | b | a | a | a | a | c | a | d | a | a | a | a | a | a | a | a | a | d | a | a | c | a |
| ICMP4010.2 | 290-300 | a | a | a | a | a | c | a | b | a | b | a | a | a | a | a | a | d | b | b | a | a | c |
| ICMP4014 | 210-220 | a | c | a | - | a | b | b | - | - | a | a | a | a | - | c | - | a | d | c | a | c | a |

Similar alleles are coded with same letter among the genotypes.
