# Supplementary material for: Development and mapping of Simple Sequence Repeat markers for pearl millet from data mining of Expressed Sequence Tags
Source: BMC Plant Biol. 2008 Nov 27;8:119. doi: 10.1186/1471-2229-8-119 (PMC2632669; doi:10.1186/1471-2229-8-119)
Supplement: Additional file 3 — Polymorphism information of genomic SSRs on parental lines of pearl millet mapping populations. [file 1471-2229-8-119-S3.doc]

Additional file 3. Polymorphism information of genomic SSRs on parental lines of pearl millet mapping populations

| Primer pair | Approximate product size (bp) | PRLT 2/89-33 | H 77/833-2 | ICMB 841-P3 | 863B-P2 | Tift 23D2B1-P1-P5 | WSIL-P8 | PT 732B-P2 | P1449-2-P1 | LGD 1-B-10 | ICMP 85410-P7 | 81B-P6 | ICMP 451-P8 | ICMP 451-P6 | H 77/833-2-P5(NT) | W 504-1-P1 | P310-17-Bk | IP 18293-P152 | Tift 238D1-P158 | ICMB 90111-P6 | ICMB 89111-P6 | IPC 804 | 81B-P8 |
| --- | --- | --- | --- | --- | --- | --- | --- | --- | --- | --- | --- | --- | --- | --- | --- | --- | --- | --- | --- | --- | --- | --- | --- |
| CTM01 | 540-550 | b | b | a | b | a | b | b | a | a | b | a | b | b | b | b | b,c | a | b | b | b |  | - |
| CTM02 | 160-250 | b | a,a1,b | - | a,a1 | a,a1 | a,a1 | a,a1 | - | b | a,a1 | a | a | a,a1 | a,a1,b | a,a1 | a1 | a,a1 | a1 | a1 | a1,b | a1,b | a1,b |
| CTM03 | 220-230 | b | a | a | c | a | a | a | b | c | c | a | a | a | a | c | b,c | a | a | a | a | c | b |
| CTM08 | 250-260 | a,b | a,a1 | a,a1 | a | a,a1 | a | a | a,a1 | a | a,a1 | a,a1 | a,a1 | a,a1 | a,a1 | a,a1 | a,a1 | a,a1 | a,a1 | a,a1,b | a,a1 | a,a1 | a,a1,b |
| CTM10 | 180-190 | c | b | d | d | a | e | d | a | e | d | d | d | d | a | d | e | e | e | c | e | f | e |
| CTM12 | 305-315 | b | c | c | b | a | b | c | a | c | c | c | a | a | c | c | a | c | b | c | c | c | c |
| CTM21 | 210-280 | g | b | a | d | a | h | e | h | e | c | a | c | - | b | h | h | f | h | b | - | c | g |
| CTM25 | 240-250 | d | - | d | c | a | d | d | d | a | c | f | - | d | c | d | e | e | d | d | - | b | c |
| CTM26 | 200 | a | a | a | a | a | a | a | a | a | a | a | a | a | a | a | a | a | a | a | a | a | a |
| CTM27 | 230-260 | a,c | c | c | b | a | c | c | c | a | c | c | a | a | a | c | a | c | b | b | b | c | - |
| CTM56 | 165 | a | a | a | a | a | a | a | a | a | a | a | a | a | a | a | a | a | a | a | a | a | a |
| CTM57 | 295-300 | a | a | a | a | - | - | a | a | a | a | a | a | a | a | a | a | - | a | b | a | a | a |
| CTM59 | 190 | a | a | a | a | a | a | a | a | a | a | a | a | a | a | a | a | a | a | a | a | a | a |
| CTM60 | 220 | a | a | a | a | a | a | a | a | a | a | a | a | a | a | a | a | a | a | a | a | a | a |

Similar alleles are coded with same letter among the genotypes.
