# Supplementary material for: Development and mapping of Simple Sequence Repeat markers for pearl millet from data mining of Expressed Sequence Tags
Source: BMC Plant Biol. 2008 Nov 27;8:119. doi: 10.1186/1471-2229-8-119 (PMC2632669; doi:10.1186/1471-2229-8-119)
Supplement: Additional file 4 — Positions of rice homologs of mapped ESTs and their putative functions. [file 1471-2229-8-119-S4.doc]

Additional file 4. Positions of rice homologs of mapped ESTs and their putative functions

| SSR loci | PM EST ID^1^ | PM LG^2^ | Homologous rice sequence ID | Rice chr^3^ | Correspondence^4^ | Putative annotation of Rice homolog |
| --- | --- | --- | --- | --- | --- | --- |
| *Xicmp*3002 | CD724823 | 6 | No hits |  |  |  |
| *Xicmp*3017 | CD724750 | 1 | AP003749, AC137616, AP002070, AC082644 | 7, 5,  1, 3 | Yes (R5L) | putative low temperature and salt responsive protein, gene P0511C01.16, similar to low temperature and salt responsive protein LTI6A GI : 4039153 |
| *Xicmp*3024 | CD724892 | 6 | AP003269, P005686, AP003825, AP004843 | 1, 9,  7, 2 | Yes (R1L) | putative photosystem II protein reaction center W, putative *Avr9*/*Cf-9* rapidly elicited protein 141, putative sex determination protein tasselseed 2 |
| *Xicmp*3025 | CD724352 | 2 | AP005802, AP003052, AP004117, AP003913, AC105745 | 9, 1,  2, 8, 10 | No | putative phosphoenolpyruvate carboxylase, phosphoenolpyruvate carboxylase |
| *Xicmp*3027 | CD724428 | 5 | No hits |  |  |  |
| *Xicmp*3031 | CD725489 | 2 | AC134238 | 3 | No | not annotated |
| *Xicmp*3032 | CD725922 | 1 | AP003749, AC137616, AP002070 | 7, 5,  1 | Yes (R5L) | putative low temperature and salt responsive protein |
| *Xicmp*3038 | CD724894 | 6 | No hits |  |  |  |
| *Xicmp*3043 | CD724407 | 7 | AP003828 | 7 | Yes (R7L) | not annotated |
| *Xicmp*3045 | CD724462 | 2 | AC138004 | 3 | Yes (R3L) | not annotated |
| *Xicmp*3048 | CD724492 | 7 | No hits |  |  |  |
| *Xicmp*3050 | CD724749 | 6 | AP002482 | 1 | Yes (R1L) | putative CBL-interacting protein kinase 1 |
| *Xicmp*3058 | CD725199 | 6 | AP002487, AP003046 | 1 | Yes (R1S) | not annotated |
| *Xicmp*3066 | CD726121 | 7 | AP004265, AP004275 | 7 | Yes (R7L) | translation initiation factor 5A |
| *Xicmp*3078 | CD726702 | 5 | AP005321, AC087542, AP004046, AC104854, AP005731, AL73160, AC105364 | 9, 10, 2, 11, 8, 4,  3 | Yes (R10L) | pseudogene, sinapyl alcohol dehydrogenase, contains similarity to mannitol dehydrogenase (NAD-dependent mannitol dehydrogenase), putative cinnamyl-alcohol dehydrogenase |
| *Xicmp*3080 | CD724750 | 1 | AP004023, L606595, AL713934 | 3, 4, 12 | No | glycosyl transferase-like protein, beta-1,4-mannosylglycoprotein 4-beta-N-acetylglucosaminyltransferase activity |
| *Xicmp*3085 | EB411016 | 1 |  |  |  |  |
| *Xicmp*3086 | EB410970 | 6 | No hits |  |  |  |
| *Xicmp*3088 | EB411043 | 1 | No hits |  |  |  |
| *Xicmp*3092 | NA | 7 | AP005167 | 7 | No | putative thioredoxin |
| *Xicmp*3093 | NA | 5 |  |  |  |  |

^1^Pearl millet EST Genbank ID; ^2^Pearl millet linkage group; ^3^Mapped rice chromosomes; ^4^Corresponding rice (R) chromosomes’ long (L) or short (S) arm
