# Supplementary material for: Development and mapping of Simple Sequence Repeat markers for pearl millet from data mining of Expressed Sequence Tags
Source: BMC Plant Biol. 2008 Nov 27;8:119. doi: 10.1186/1471-2229-8-119 (PMC2632669; doi:10.1186/1471-2229-8-119)
Supplement: Additional file 5 — Details of pearl millet inbred lines used for polymorphism testing. [file 1471-2229-8-119-S5.doc]

Details of pearl millet inbred lines used for polymorphism testing

| **Genotype** | **Origin** | **Characteristics** | **Reference*** |
| --- | --- | --- | --- |
| H 77/833-2 | Bred at CCS Haryana Agricultural University by selfing and selection within a Rajasthani landrace population | Tolerant to seedling heat stress, small grain size, narrow leaf blades, thin stems, thin panicles, profuse basal and nodal tillering | Kapoor *et al*. [1]; Yadav *et al*. [2] |
| PRLT 2/89-33 | Inbred derived from the ICRISAT Bold Seeded Early Composite, elite breeding population based predominantly on *Iniadi* landrace germplasm from West Africa | Sensitive to seedling heat stress; large grain size, broad leaf blades, thick stem, thick panicle, very little tillering | Yadav *et al*. [2]; Witcombe and Soman [3]; Andrews and Anand Kumar [4]; |
| ICMB 841-P3 | Bred at ICRISAT by pure-line selection for downy mildew resistance in seed lot of elite maintainer line MS 5141B | Small seed size, narrow leaf blades, thin panicles, and thin stems; poor combining ability for terminal drought tolerance | Singh *et al*. [5]; Yadav *et al*. [6]; Bidinger *et al*. [7]; Hash *et al*. [8] |
| 863B-P2 | Bred at ICRISAT from *Iniadi* landrace material from Togo by selfing and selection | Large grain size, broad leaf blades, thick stems, and thick panicles, downy mildew resistant, good combining ability for terminal drought tolerance and good stover quality | Andrews and Anand Kumar [4]; Yadav *et al*. [6]; Bidinger *et al*. [7]; Hash *et al*. [8] |
| Tift 23D_2_B_1_-P1-P5 | Bred at the Coastal Plain Experiment Station by introducing the *d*_2_ dwarfing gene into the genetic background of elite seed parent maintainer line Tift 23B_1_ | Reference genotype for pearl millet genomics; *d*_2_ dwarf, *hl* hairy leaf blade, *Hm* hairy leaf margin; maintainer for A_1_ cytoplasmic-genetic male-sterility system, highly susceptible to downy mildew, rust, smut and ergot | Burton [9, 10]; Liu *et al*. [11]; Allouis *et al*. [12] |
| WSIL-P8 | Selection from IP 18292, genetic stock developed in ICRISAT-Patancheru from a complex cross of diverse parental materials | *d*_2_ dwarf, *gl* glossy seedling, *ws* white leaf sheath, long panicles; *Rsg*_1_ major gene for downy mildew resistance on PM LG2; | Liu *et al*. [11]; Appa Rao *et al*. [13, 14]; Singh *et al*. [15]; Singh and Talukdar [16] |
| PT 732B-P2 | Tamil Nadu Agricultural University (TNAU) | Agronomically elite, *d_2_* dwarf, photoperiod-sensitive | Appadurai *et al*. [17] |
| P1449-2-P1 | Selection (IP 21168) made at ICRISAT-Patancheru from germplasm accession IP 5853 originated from Senegal | Tall, downy mildew and rust resistant | Singh *et al*. [15]; Singh [18] |
| LGD 1-B-10 | Derivative of (B70 × Tift 756)-1-4-5 based on *Iniadi* landrace germplasm from Togo (B70) and a breeding line from the Coastal Plain Experiment Station, USA (Tift 756) | Downy mildew susceptible, *d*_2_ dwarf, *e*_1_ photoperiod-insensitive early flowering; large grain size; non-standard chromosome complement (translocation homozygote) | Liu *et al*. [11]; Hash [19]; Jones *et al*. [20, 21] |
| ICMP 85410-P7 | Derivative of (IPC 165 × IPC 220)-64 based on a germplasm from Uganda, Mali and Nigeria | Late-flowering *d*_2_ dwarf, long compact panicle, physiologic leaf spot, fertility restorer for A1 cytoplasmic-genetic male-sterility system; moderately resistant to downy mildew | Liu *et al*. [11]; Jones *et al*. [20, 21] ; Talukdar *et al*. [22] |
| 81B-P6 | Downy mildew resistant selection from gamma radiation-treated Tift 23D_2_B_1_ | *d*_2_ dwarf with *hl* hairy leaf blades; later flowering than Tift 23BD; long, thin, short-bristled panicles; maintainer of A_1_ cytoplasmic-genetic male-sterility system; downy mildew resistance now largely ineffective in India | Anand Kumar *et al*. [23] |
| ICMP 451-P8 | Derived from LCSN 72-1-2-1-1, a selection made in Upper Volta from the ICRISAT Center Late Composite | Tall having long-bristled semi-compact panicles, smooth leaf blades and amber-grey colored globular seeds, moderately resistant to downy mildew; slow-rusting | Anand Kumar *et al*. [24] |
| ICMP 451-P6 | Derived from LCSN 72-1-2-1-1, a selection made in Upper Volta from the ICRISAT Center Late Composite | Tall having bristled semi-compact panicles, smooth leaf blades and amber-grey colored globular seeds, moderately resistant to downy mildew; slow-rusting; downy mildew resistance donor used for marker-assisted improvement of H 77/833-2 | Anand Kumar *et al*. [24]; Breese *et al*. [25]; Hash *et al*. [26] |
| H 77/833-2-P5(NT) | Sub-selection of H 77/833-2, which was originally bred at CCS Haryana Agricultural University from a Rajasthani landrace population | Early to flower, medium height with profuse tillering; susceptible to downy mildew and rust; conical-shaped panicle without bristles; thicker panicles and larger grains than original H 77/833-2 | Breese *et al*. [25] |
| W 504-1-P1 | Inbred breeding line from the Indian Agricultural Research Institute, New Delhi, based on germplasm from Northern India | Tall, good early seedling vigor, downy mildew susceptible | Sheoran and Govila [27] |
| P310-17-Bk | Bred at ICRISAT-Patancheru by selfing and selection within germplasm accession IP 6329 originating from Mali, West Africa | Tall, downy mildew resistant | Singh *et al*. [15] |
| IP 18293-P152 | Isolated at ICRISAT-Patancheru from a segregating population from the cross IP 10399 × IP 10729 | *P* purple-foliaged, *d*_2_ dwarf, highly resistant to downy mildew | Appa Rao *et al*.[14]; Azhaguvel *et al*. [28] |
| Tift 238D1-P158 | Developed at the Coastal Plain Experiment Station, Tifton, USA | Downy mildew-susceptible, green-foliaged, *d*_1_ dwarf line carrying carry a single dominant gene for fertility restoration in the A_1_ cytoplasmic-genetic male-sterility system | Azhaguvel *et al*. [28]; Burton [29] |
| ICMB 89111-P6 | Downy mildew susceptible selection from within ICMB 89111, which was bred at ICRISAT-Patancheru from the cross 843B × (Gero New Source × Saria Synthetic-48-40-4)-1-9-8 | Semi-dwarf (*d_2_*), high tillering, moderately photoperiod-sensitive and downy mildew susceptible; maintainer of the A_1_ cytoplasmic-genetic male-sterility system | Rai and Rao [30]; Gulia *et al*. [31] |
| ICMB 90111-P6 | Downy mildew resistant selection from ICMP 423 (IPC 94), which has the pedigree EC-S3-211-1-2 | Tall, high tillering, moderately photoperiod-sensitive and downy mildew resistant; weak restorer of A_1_ and maintainer of A_egp_ cytoplasmic-genetic male-sterility systems | Rai and Rao [30]; Gulia *et al*. [31] |
| 81B-P8 | Downy mildew resistant selection from gamma radiation-treated Tift 23D_2_B_1_ | Semi-dwarf (*d_2_*); hairy leaf blades (*hl*), leaf margins (*Hm*) and leaf sheaths (*hsh*); short-bristled panicles; maintainer of A_1_ and A_4_ cytoplasmic-genetic male-sterility systems | Anand Kumar *et al*. [23]; Yadav [32] |
| IPC 804 | Breeding line developed at ICRISAT-Patancheru by crossing S 10LB (a long-bristled inbred line developed at Punjab Agricultural University, Ludhiana, India from a Serere Composite) and LCSN 1225-6-3-1 (a progeny identified at Kamboinse, Burkina Faso from ICRISAT’s Late Composite) | Tall (non-*d_2_*), with non-hairy leaves and stem, and presence of long panicle bristles; triple-restorer of the A_1_, A_4_ and A_egp_ CMS systems, with profuse pollen producing capacity | Yadav [32] |

*References:

1. Kapoor RL, Kakkar PS, Khairwal IS, Bainiwal CR, Nijhawan DC, Yadav, HP. **Bajra hybrid HHB 67—a major break through.** *Haryana Farming* 1988, 18:17-21.
2. Yadav RS, Hash CT, Bidinger FR, Cavan GP, Howarth CJ: **Quantitative trait loci associated with traits determining grain and stover yield in pearl millet under terminal drought-stress conditions.** *Theor Appl Genet* 2002, 104:67-83.
3. Witcombe JR, Soman P: **Selection for seedling emergence using a glasshouse test in pearl millet.** *Plant Breed* 1992, 108:75-80.
4. Andrews DJ, Anand Kumar K: **Use of the West African pearl millet landrace Iniadi in cultivar development.** *Plant Genet Resour Newslett* 1996, 105:15-22.
5. Singh, SD, Singh P, Rai KN, Andrews DJ: **Registration of ICMA 841 and ICMB 841 pearl millet parental lines with A1 cytoplasmic–genic male sterility system.** *Crop Sci* 1990, 30:1378.
6. Yadav RS, Hash CT, Bidinger, FR, Devos KM, Howarth CJ: **Genomic regions associated with grain yield and aspects of post-flowering drought tolerance in pearl millet across stress environments and tester background.** *Euphytica* 2004, 136:265-277.
7. Bidinger FR, Nepolean T, Hash CT, Yadav RS, Howarth CJ: **Quantitative trait loci for grain yield in pearl millet under variable postflowering moisture conditions**. *Crop Sci* 2007, 47:969-980.
8. Hash CT, Bhasker Raj AG, Lindup S, Sharma A, Beniwal CR, Folkertsma RT, Mahalakshmi V, Zerbini E, Blümmel M: **Opportunities for marker-assisted selection (MAS) to improve the feed quality of crop residues in pearl millet and sorghum.** *Field Crop Res* 2003, 84:79-88.
9. Burton, GW: **Pearl millets Tift 23DA and Tift 23DB released.** *Georgia Agric Res* 1967, 9:6.
10. Burton, GW: **Registration of pearl millet inbreds Tift 23B_1_, Tift 23A_1_, Tift 23DB_1_, and Tift 23D2A_1_ (Reg. Nos. PL 1, PL 2, PL 3 and PL 4).** *Crop Sci* 1969, 9:397.
11. Liu CJ, Witcombe JR, Pittaway TS, Nash M, Hash CT, Busso CS, Gale MD: **An RFLP-based genetic map of pearl millet (*Pennisetum* *glaucum*).** *Theor Appl Genet* 1994, 89:481-487
12. Allouis S, Qi X, Lindup S, Gale MD, Devos KM: **Construction of a BAC library of pearl millet, *Pennisetum glaucum*.** *Theor Appl Genet* 2001, 102:1200-1205
13. Appa Rao, S, Mengesha MH, Reddy CR, Rao YS: **Genetics of white sheath and bleached leaf mutants in pearl millet.** *J Hered* 1990, 81:159-162.
14. Appa Rao S, Singh SD, Mengesha MH, Reddy KN, Rao SN: **Development and characterization of genetic stocks in pearl millet (*Pennisetum glaucum*) resistant to downy mildew (*Sclerospora graminicola*).** *Indian J Agric Sci* 1996, 66:221-223.
15. Singh SD, Wilson JP, Navi SS, Talukdar BS, Hess DE, Reddy KN: **Screening techniques and sources of resistance to downy mildew and rust in pearl millet**. International Crops Research Institute for the Semi-Arid Tropics: Patancheru, 502 324, Andhra Pradesh, India, 1997, Information Bulletin number 48.
16. Singh SD, Talukdar BS: **Inheritance of complete resistance to pearl millet downy mildew.** *Plant Dis* 1998, 82:791-793.
17. Appadurai R, Raveendran TS, Nagarajan C: **A new male-sterility system in pearl millet.** *Indian J Agric Sci* 1982, 52:832-834.
18. Singh, SD: **Sources of resistance to downy mildew and rust in pearl millet.** *Plant Dis* 1990, 74:871-874.
19. Hash CT: **Breeding for large grain size in pearl millet (*Pennisetum americanum* (L.) Leeke).** *PhD thesis*, Cornell University, Ithaca, New York, USA, 1986, 242 pp.
20. Jones ES, Liu CJ, Gale MD, Hash CT, Witcombe JR: **Mapping quantitative trait loci for downy mildew resistance in pearl millet.** *Theor Appl Genet* 1995, 91:448-456
21. Jones ES, Breese WA, Liu CJ, Singh SD, Shaw DS, Witcombe JR: **Mapping quantitative trait loci for resistance to downy mildew in pearl millet: Field and glasshouse screens detect the same QTL.** *Crop Sci* 2002, 42:1316-1323
22. Talukdar BS, Prakash Babu PP, Rao AM, Ramakrishna C, Witcombe JR, King SB, Hash CT: **Registration of ICMP 85410: Dwarf, downy mildew resistant, restorer parental line of pearl millet.** *Crop Sci* 1998, 38:904-905.
23. Anand Kumar K, Andrews DJ, Jain RP, Singh SD: **ICMA-1 (*sic*) and ICMB-1 (*sic*) pearl millet parental lines with A_1_ cytoplasmic-genic male-sterility system.** *Crops Sci* 1984, 24:832.
24. Anand Kumar K, Rai KN, Andrews DJ, Talukdar BS, Singh SD, Rao AS, Babu PP, Reddy BP: **Registration of ICMP 451 parental line of pearl millet.** *Crop Sci* 1995, 35:605.
25. Breese WA, Hash CT, Devos KM, Howarth, CJ: **Pearl millet genomics – an overview with respect to breeding for resistance to downy mildew.** In: *Sorghum and Millets Pathology 2000*. Edited by Leslie JF. Ames, Iowa, Iowa State Press 2002, 243-246
26. Hash CT, Sharma A, Kolesnikova-Allen MA, Singh SD, Thakur RP, Bhasker Raj AG, Ratnaji Rao MNV, Nijhawan DC, Beniwal CR, Sagar P, Yadav HP, Yadav YP, Srikant, Bhatnagar SK, Khairwal IS, Howarth CJ, Cavan GP, Gale MD, Liu C, Devos KM, Breese WA, Witcombe JR: **Teamwork delivers biotechnology products to Indian small-holder crop-livestock producers: Pearl millet hybrid “HHB 67 Improved” enters seed delivery pipeline.** *J SAT Agric Res* 2006, 2(1): http://www.icrisat.org/journal/bioinformatics/v2i1/v2i1teamwork.pdf
27. Sheoran RK, Govila OP: **Combining ability analysis of downy mildew resistant and susceptible lines of pearl millet.** *Crop Improv* 1996, 23:57-60.
28. Azhaguvel P, Hash CT, Rangasamy P, Sharma A: **Mapping the *d*_1_ and *d*_2_ dwarfing genes and purple foliage color locus *P* in pearl millet.** *J Hered* 2003, 94:155-159.
29. Burton GW: **Pearl millet breeding**. *Sols Africans* 1966, 11:39-42.
30. Rai, KN, Rao AS: **Registration of pearl millet parental lines ICMA 89111 and ICMB 89111.** *Crop Sci* 1998, 38:1412-1413.
31. Gulia SK, Hash CT, Thakur RP, Breese WA, Sangwan RS: **Mapping new QTLs for improvement of downy mildew resistance in pearl millet.** In: *Crop Production in Stress Environments: Genetic and Management Options*. Edited by Singh DP, Tomar VS, Behl RK, Upadhyaya SD, Bhale MS, Khare B. Jodhpur, Rajasthan, India, Agrobios International, 2007, 373-386
32. Yadav D: **Genetics of cytoplasmic-nuclear male sterility and identification of molecular markers of fertility restoration in pearl millet (*Pennisetum glaucum* (L.) R. Br).** 2005, *PhD thesis*. Chaudhary Charan Singh Haryana Agricultural University, Hisar, Haryana, India.
